# Comparison of Groundwater Level Models Based on Artificial Neural Networks and ANFIS

**DOI:** 10.1155/2015/742138

**Published:** 2015-11-23

**Authors:** Nevenka Djurovic, Milka Domazet, Ruzica Stricevic, Vesna Pocuca, Velibor Spalevic, Radmila Pivic, Enika Gregoric, Uros Domazet

**Affiliations:** ^1^University of Belgrade, Faculty of Agriculture, Nemanjina 6, Zemun, 11000 Belgrade, Serbia; ^2^Electric Power Industry of Serbia, Vojvode Stepe 412, 11000 Belgrade, Serbia; ^3^The Institute of Forestry of Montenegro, Novaka Miloseva 10/II, 81000 Podgorica, Montenegro; ^4^Institute of Soil Science Belgrade, Teodora Drajzera 7, 11000 Belgrade, Serbia; ^5^Military Academy, Generala Pavla Jurisica Sturma 33, 11000 Belgrade, Serbia

## Abstract

Water table forecasting plays an important role in the management of groundwater resources in agricultural regions where there are drainage systems in river valleys. The results presented in this paper pertain to an area along the left bank of the Danube River, in the Province of Vojvodina, which is the northern part of Serbia. Two soft computing techniques were used in this research: an adaptive neurofuzzy inference system (ANFIS) and an artificial neural network (ANN) model for one-month water table forecasts at several wells located at different distances from the river. The results suggest that both these techniques represent useful tools for modeling hydrological processes in agriculture, with similar computing and memory capabilities, such that they constitute an exceptionally good numerical framework for generating high-quality models.

## 1. Introduction

The physical dependency between climatic and hydrological quantities is highly complex and nonlinear in nature. Numerous parameters that describe this physical dependency are largely unknown or difficult to measure. Although physical models are widely used to model groundwater flow in the vadose zone [1], they exhibit a significant shortfall in that they require accurate characterization and quantification of physical properties and mutual dependencies within the system under consideration.

On the other hand, there are parametric models based on so-called data driven techniques. They can serve as an alternative to physical models, to model different water table scenarios or reconstruct long periods of missing observations, in order to explain the correlations between the quantities that have been measured. Compared to physical models, the advantage of parametric models is that they do not require data on initial and boundary conditions, or aquifer characteristics, and are therefore useful when there is limited knowledge of hydrological and hydrogeological parameters.

One such parametric model is the artificial neural network (ANN), widely used in science and engineering. ANN is an information processing system that features learning, memorizing, and generalizing on the basis of training data. ANN are an important tool in estimation in hydrology [2, 3].

In groundwater studies, the advantage of ANN models is that they can be used with a high level of accuracy to improve management strategies for a broad spectrum of groundwater problems, relating to both quantitative and qualitative aspects [4–12]. Comparisons with other parametric models have shown that ANN models, regardless of whether they produce better or worse results than other models, can be used to very accurately predict groundwater levels on given localities [13–15]. Many authors have used climate data as input parameters to model the water table: precipitation [16] and precipitation and temperature [17–20]. Uddameri [21] showed that in the deeper sections of the Evangeline formation of the Gulf coastal aquifer there was no effect of climate parameters on the water table.

Soft computing techniques, such as the fuzzy inference system (FIS), have also been effectively applied to forecast groundwater levels. Alvisi et al. [22] used fuzzy logic to model the dependency between precipitation and groundwater levels in Italy. The combination of ANN and FIS into an adaptive neurofuzzy inference system (ANFIS) led to the development of new computing techniques, applied in hydrology to model hydrological phenomena as a function of precipitation [23–25]. The second model used in this research was ANFIS (adaptive neurofuzzy inference system or adaptive network-based fuzzy inference system). Neural network-driven fuzzy reasoning first appeared in the literature at the end of the past century. Given that such models were described in the works of Takagi, and Sugeno and Kang [26–28], they are often referred to as TSK models. The basic idea of this approach is to use a membership function to compute the fulfillment of a prerequisite for an activity or decision and then to quantify the activity or decision through the neural network output.

There are a number of works in the literature that address the application of ANFIS to model various physical processes in hydrology and agriculture, or which compare the efficiency of ANFIS with neural networks. Tutmez et al. [29] used ANFIS to model groundwater conductivity based on positive ion concentrations in solution and demonstrated that modeling was possible even in cases where little groundwater information was available. Ponnambalam et al. [30] showed that in water resource optimization ANFIS had advantages over other models based on multiple regression analysis. Singh and Deo [31] compared different forms of neural networks and ANFIS for river flow modeling and concluded that the use of a complex neural network was necessary to model hydrological phenomena, due to their intricate nature.

Talebizadeh and Moridnejad [32] compared ANFIS and ANN in modeling water levels of lakes, using precipitation, evapotranspiration, and inflow as input data, and concluded that ANFIS yielded better results. Other authors [33, 34] reported similar findings after modeling the dependency between precipitation and groundwater levels and comparing ANFIS and RBF (Radial Basis Function) models used to forecast seasonal water tables. Shirmohammadi et al. [35] modeled groundwater applying several data-driven techniques, including system identification, time series, and ANFIS models, and demonstrated the advantages of ANFIS over other approaches when they modeled nonlinear time series. Moosavi et al. [36] assessed hybrid ANN and ANFIS models, where wavelet transform was applied to the data used as inputs into these models. They analyzed the ANFIS architecture and determined the optimal number of neurons in the hidden layer, forecasting groundwater levels for different prediction periods. With regard to daily water table forecasts, Affandi and Watanabe [37] demonstrated that both ANFIS and ANN models can be used with a high level of precision.

However, despite the fact that the above references suggest a slight advantage of either ANFIS or ANN, any comparison of the efficiency of these modeling tools is a very delicate matter for several reasons. The first is that ANFIS supports explicit entry of* a priori* knowledge or expert judgment into the model through the selection and number of membership functions for each individual input variable. Although this appears to be an indisputable advantage of ANFIS, it is seldom exploited because it is not clear to designers what initial data clustering really means, and such clustering is the starting point of ANFIS training. On the other hand, the ANN training technique often involves backpropagation errors, while in most ANFIS training a hybrid technique is applied, which uses the least squares method in addition to backpropagation errors. This advantage also speaks in favor of ANFIS. Further, it is much easier to define the complexity of a system in the case of ANN. By selecting the number of hidden layers and the number of nodes within them, the user directly determines the modeling power and, consequently, the complexity of the network. On the other hand, whenever a multivariate system is addressed, the complexity of ANFIS can be adjusted by the selection of the cluster radius used in initial subtractive clustering. Although the physical sense of this parameter is intuitively clear, the user cannot know in advance which value of this parameter will result in a certain model complexity.

Many authors have studied the problem of groundwater modeling, but they largely used climate parameters (precipitation, temperature, and evaporation) as input parameters for the ANN or ANFIS model [17–20, 34]. Very few authors have modeled the impact of river stages, which proved to be the dominant factor in our study area; a correlation analysis revealed that the impact of climate parameters was negligible. Also, available literature does not include reports on comparative analysis of ANN and ANFIS, as applied to model the effect of river stages on groundwater levels in agricultural areas. Additionally, what is missing in available literature is that authors generally disregard the link between the number of available measured data points and the complexity of the structure used for modeling. As a result, the number of parameters that need to be adjusted on the model might be several times greater than the number of available observations, and the simple conclusion is that the model is good even though such a conclusion is not statistically justified. The research reported in this paper took into account that the compared ANN and ANFIS should have comparable levels of complexity, measured by the number of parameters that needed to be adjusted. Only such a comparison seemed reasonable.

In view of the above, two stochastic models were selected for the present research: ANFIS and feedforward neural network, with the objective of assessing the applicability of these techniques to water table forecasts and to compare them to actual monitoring data collected from Danube's riparian lands. Special attention was devoted to the comparison of these two approaches from a training complexity perspective, which addressed feeding of* a priori* knowledge to the model and how demanding the size of the training set was. Three observation points, at different distances from the river, were selected for analysis, to demonstrate to what extent the models were suitable for monthly forecasts of depths-to-water table in different parts of the alluvial plain.

## 2. Study Area

The riparian lands of the Danube in Serbia are spacious and very important to Serbia's agricultural sector. Under natural conditions, when the stages of the Danube were below the water table of the “first” aquifer in the protected floodplain, and of the upper terrace aquifer, the river drained groundwater. However, these natural conditions were altered after the Đerdap 1 (Iron Gate 1) and Đerdap 2 (Iron Gate 2) hydropower and navigation systems were built on the Danube at rkm 943 and rkm 862.8, respectively, on a reach of the Danube that defines the border between Serbia and Romania. Even though the first turbines of Iron Gate 1 HPP were placed online in 1970, the entire project was commissioned in 1972. This project altered the hydrological conditions in the general area, as impoundment resulted in the creation of a reservoir extending as far as several hundred kilometers upstream from the dam. Given that Djerdap 1 and Djerdap 2 were built in 1970 and 1985, respectively, the previous groundwater regime was not really relevant to our research. As such, the analyzed data are from the period from 1990 to 2010. The paper mentions these two HPPs because their dams have largely altered the natural regime of the Danube.

As such, the Danube raised the water table of the “first” aquifer. Given the location of the area (alluvial plain) of the Danube and the hydrogeological and hydrodynamic conditions (dual-layer porous medium), stage fluctuations of the Danube propagate through the lower water-bearing layer, causing corresponding piezometric head changes and thus affecting the groundwater level regime of the upper semipermeable horizon. Consequently, the stage variations caused by Iron Gate 1 propagate inland. Drainage systems along the river maintain a certain piezometric head regime in the water-bearing layer and control Danube's impact. This eliminates the adverse effect of the reservoir, as well as that of naturally high stages, on the riparian farmland. However, in addition to Iron Gate 1 HPP (1972), Iron Gate 2 HPP was built in 1977–1985. and plans call for another HPP, Iron Gate 3. This will increase the complexity of hydrological conditions and mutual influences of the hydropower projects, such that water table predictions are becoming increasingly important to the riparian farmland. Reliable water table forecasts will ensure timely preparation for the operation of pumping stations and other drainage system facilities, to support maintenance of prescribed groundwater levels and unhindered agricultural production.

**Figure 1:**
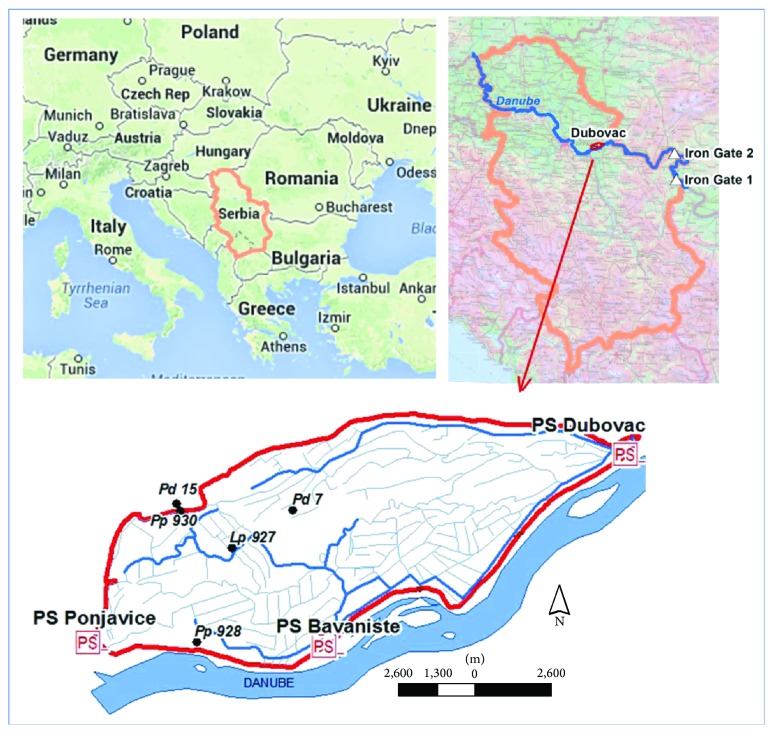
Location map of the study area.

The study area lies between the town of Kovin and the village of Dubovac (Figure 1). It occupies a land area of 92.41 km^2^, from rkm 1150 to rkm 1175 of the Danube, between 44° 48′ 20′′ and 44° 41′ 11′′ N and 20° 58′ 00′′ and 21° 12′ 16′′ E. It is located in South Banat region, in the Province of Vojvodina. The southern edge of the study area borders on the Danube for 25 km. To the north, the study area borders on a loess terrace to the village of Gaj and to Deliblato Sands, gradually descending towards the Danube at Dubovac. The entire study area is situated within Danube's alluvial plain. Altitudes vary from 9.7 to 86 m above sea level, mostly 60–70 m a.s.l. The majority of the relief features a 2–4‰ slope. Under natural conditions, when Danube's stages were below the water table of the first aquifer in the protected floodplain, as well as that of the upper terrace, the river drained groundwater. A certain piezometric head regime in the water-bearing layer is maintained by drainage systems, to ensure proper farming conditions. Since commissioning of the Iron Gate 1 project, the depths-to-water table of the “first” aquifer has varied from 0.37 to 1.56 m (minimum) and from 1.07 to 3.17 m (maximum). The average annual fluctuations are about 0.5 m. The highest water tables are generally recorded in January, February, and March, and the lowest in August and September.

The so-called Main Outer Channel crosses the entire study area and ends at a pumping station in Dubovac. There are also two primary channels: one runs parallel to the Danube and constitutes the “first line of defense,” and the other crosses the middle of the study area, where the terrain is naturally the lowest, and ends at a pumping station in Bavaniste.

There are three lines of drainage channels:

- First line: along an embankment, providing protection from the Danube.
- Second line: regulating groundwater levels within the area.
- Third line: providing protection against inflow from the hinterland.

One well was selected in each part of the study area, for which the water table was modeled. The objective was to determine to what extent the distance from the river affected water table forecasts and if there were any significant differences in the applicability of stochastic models.

## 3. Methods

### 3.1. Selection of Input and Output Variables

As previously stated, the input parameters were used to predict groundwater levels at three wells. The selection of these wells was not arbitrary. Namely, they were located in three separate parts of the study area: the first was close to the Danube (Pp 928), at a distance of 350 m; the second was located in the central part (Lp 927), at a distance of 4080 m; and the third was in the protected floodplain but farthest from the Danube (Pp 930), at a distance of 4850 m. They were also representative of the three lines of drainage: (1) closest to the embankment along the Danube, where the stage has the greatest effect on the depth-to-water table; (2) inside the study area; and (3) within the zone of influence of groundwater flows from the hinterland. The objective of selecting these particular wells was to assess whether the above-mentioned models could effectively be used for the entire drainage area, regardless of the distribution of the wells.

The design of the network architecture was preceded by the selection of significant input variables. This selection is extremely important in model development, given that a large number of input variables slow down the model and it is also not always possible to obtain statistically significant data on the interactions of individual variables. Clearly not all the potentially considered physical quantities have the same influence on the output variable. The starting point in the selection of input variables was expert judgment, or* a priori* knowledge about the physical processes to be modeled. However, expert judgment is frequently prone to subjective assessment. In view of the fact that dependencies between quantities are often highly complex and that the relations between the quantities to be modeled are not straightforward or cannot be explicitly defined, usually the best approach is to resort to a combination of expert judgment and analytical methods. Some authors [38–41] have used analytical techniques, such as cross-correlation and autocorrelation analyses, to determine the linear correlation between some of the considered variables.

A correlation analysis was undertaken in this research as well, taking physical quantities for which it was assumed, based on experience, that they might have an effect on the water table: past groundwater levels, water levels in nearby wells, Danube's stages, precipitation, evapotranspiration, and air temperature. Two neighboring piezometers were used in the analysis; these were the closest piezometers for which a continuous time series was available and which also applied to the selected piezometers. Given that the geological conditions were the same across the study area, this selection was based on the least distance between the piezometers. All quantities were on a monthly basis, for the period June 1990–May 2010.

The correlation analysis served to select the pairs of physical variables between which a significant causal relationship was established. This analysis showed that there was no significant dependency between precipitation, climate parameters (air temperatures), and groundwater levels at the studied wells.  Table 1 contains some of the coefficients of correlation computed within this analysis.

The coefficients of correlation are extremely low, or very close to zero. Namely, the theory is that the coefficient of correlation between two random variables can be in the interval [−1,1]. The closer the coefficient to zero, the less significant the correlation between them. In such a case the physical parameters are deemed not to correlate. If a normal distribution is assumed, the mutual independence of the parameters is also indicated.

**Figure 2:**
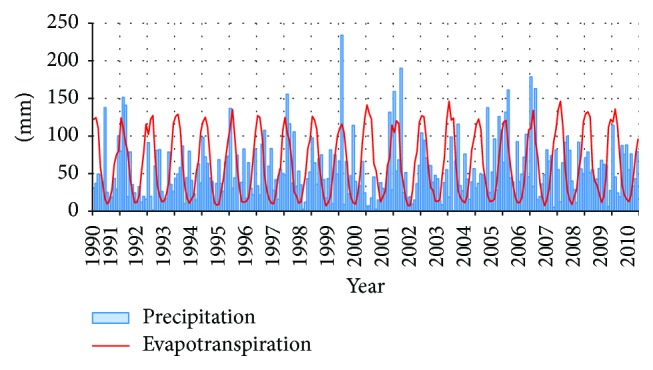
Monthly precipitation (*P*) and evapotranspiration (ETo) versus time in period 1990–2010.

Although the depth-to-groundwater was several meters from the ground surface, climate parameters on a monthly basis had no significant effect on groundwater. When measured precipitation levels were compared to the reference evapotranspiration (ETo) (Figure 2), computed by the Penman-Monteith method, [42], the heaviest precipitation was noted when the reference evapotranspiration was the highest, and evapotranspiration rates exceeded annual precipitation levels by 150 mm on average. When the effect of maximum daily temperatures was assessed, no significant dependency was noted.

**Figure 3:**
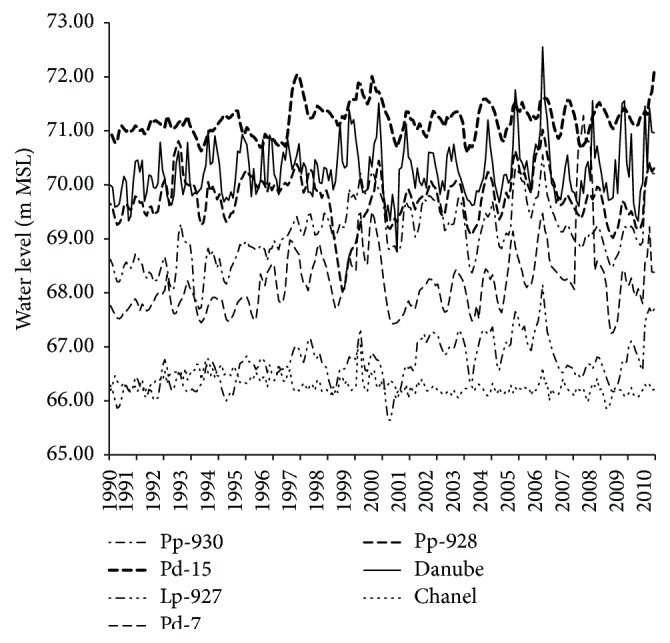
Monthly groundwater level fluctuations versus time in period 1990–2010.

On the other hand, the same approach was followed to select several quantities that had a significant effect on the water table. The correlation analysis revealed a strong causal relationship between the water depth at a given well, the water depth at surrounding wells, Danube's stage, and the water level in the main channel from which water was discharged into the Danube either gravitationally or by pumping. A regression correlation was established between Danube's stage, the water level in the channel, the water depth at the nearby wells, and the considered signal (correlation coefficient 0.287–0.623). However, since correlation analysis does not show dependency as a function of time, moving-average regression models [43, 44] were used in the present research, determining which input variables were to be included; along with the model order and delay Figure 3 shows monthly groundwater level fluctuations versus time at accessible wells, as well as the stages of the Danube and the channel. The shift in the average values of these parameters is apparent in the figure, and the fluctuation dynamic is also obvious and visually discernible.

The ARMA (autoregressive–moving-average) modelwas used to analyze the input data set, to determine the model order and delay, and to define the input set of variables as well as possible. The analysis showed that the delay had no effect on the ARMA model, and the model order was found to be 3, in the case of water levels at the given well and neighboring wells. On the other hand, the model order in the case of Danube's and the channel's stages was 0, such that the output data set was defined by *x*(*t*-1), *x*(*t*-1), *x*(*t*-1), *z*(*t*-1), *z*(*t*-1), *z*(*t*-1), *z*(*t*-1), *z*(*t*-1), *z*(*t*-1), Danube (*t*-1), and channel (*t*-1) (Table 2).

### 3.2. Design of ANN Based Model

In the present research, water table dependency was modeled using the ANN approach, which represents a generation of information processing systems that feature learning, memorizing, and generalizing on the basis of training data. Neural networks are comprised of several interconnected layers, resulting in a multilayer feedforward network. ANN enables modeling of the dependencies of certain physical quantities and of the variable whose value needs to be predicted at a certain point in time. The input layer contains the values of input variables, or those physical quantities that affect the variable to be predicted. It usually has no function other than receiving and buffering input signals. The outputs from the network are generated by the output layer. Each layer between the input layer and the output layer is referred to as a hidden layer, because it is an internal layer of the given network, which has no direct contact with the external environment. ANN can include zero to several hidden layers. A network is said to be fully connected if each output from a layer is linked with each node of the next layer. A single-layer feedforward network was constructed for this research, which produced sound water table forecasts [15, 45, 46].

**Figure 4:**
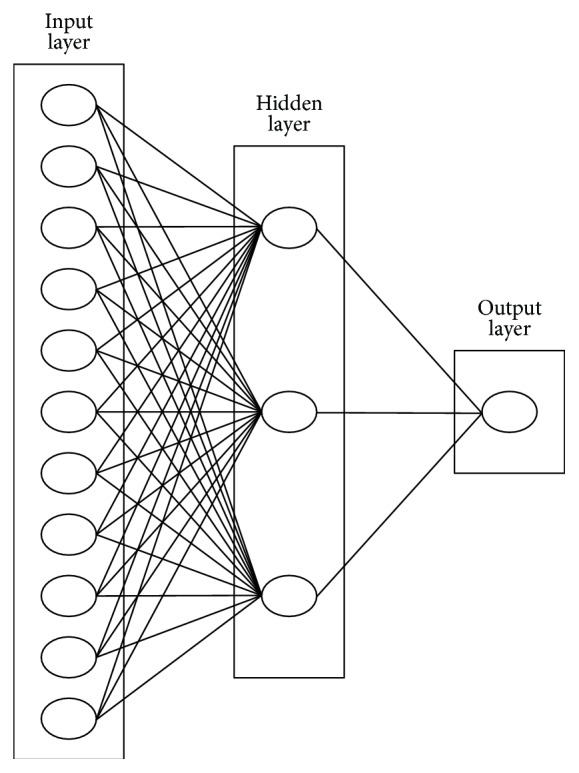
ANN architecture.

The ANN architecture commonly employed one hidden layer [13, 16, 38, 47]. The output layer had only one variable, depth-to-groundwater at the considered well, as a monthly value (Figure 4). The variables were standardized for zero mean and unit variation and then normalized for 0.1–0.9 [48]. The activation function for the hidden and output layers was a tansig and purelin function, respectively, as these proved to be the best by trial and error. In the trial and error procedure the number of nodes in the hidden layer was varied from 3 to 6, and the root mean squared error (RMSE), coefficient of determination (*R*), and coefficient of efficiency (COE) [16, 43] were calculated in each case.

The conclusion of this exercise was that as the number of nodes in the hidden layer increased, these quality criteria slightly improved (lower RMSE and higher *R* and COE), but the structure of the network and the number of parameters to be tuned increased considerably. Consequently, it was deduced that three nodes were a good compromise between modeling quality and network training complexity. Three hidden nodes are also suggested in the literature [12, 16, 18], although Sreekanth et al. [49] demonstrated that the number of nodes in the hidden layer can be far greater.

ANN training was conducted applying the Levenberg–Marquardt (LM) backpropagation error method [38, 43] where one of the following three conditions concluded the training:

1. Maximum number of iterations (1000) is achieved.
2. Required value of MSE (mean square error) criterion (0.1) is achieved.
3. The validation set MSE (mean square error) criterion increased over a certain number of consecutive iterations (6).

The purpose of the last criterion was to prevent ANN data overfitting. This approach is generally used where the network tends to memorize training examples without learning how to generalize to new situations. After the input data and the number of neurons for the input, hidden and output layers were selected, the simulation data set was divided into three groups: 60% of the data from the available time series were used for validation 1990–2002, 20% were for validation 2003–2006, and 20% were reserved for testing 2007–2010.

### 3.3. Design of ANFIS Based Model

As previously mentioned, neural network driven fuzzy reasoning systems are often referred to as TSK (Takagi-Sugeno-Kang) models. The basic idea behind such models is to use membership functions to find out if a prerequisite for an activity or decision has been fulfilled and to then quantify the activity or decision through the neural network output. These systems have been developed with the objective of solving two problems that are always present in the design of fuzzy reasoning systems. The first is the lack of a singular approach to the selection of membership functions and the determination of the parameters within them. The second is related to a lack of training functions in the autotuning of decision rules.

The most general structure of decision rules in TSK (Takagi-Sugeno-Kang) systems is

(1) Rs:IF  x=x1,…,xn  is  AsTHEN  ys=NNsx1,…,xn,s=1,…,r,

where *r* is the total number of decision rules, *A*_*s*_ is the fuzzy set which defines the prerequisite for applying the rules, where each rule has its prerequisite fuzzy set, and *NN*_*s*_( ) is the neural network whose inputs are (*x*_1_,…, *x*_*n*_) and whose output is *y*_*s*_.

One of the most commonly used TSK (Takagi-Sugeno-Kang) systems is the adaptive network-based fuzzy inference system, or ANFIS. Typical of ANFIS is that the outcomes, or the ultimate output of the reasoning system, are presented in the form of a linear combination of the causes leading to the effect. In other words, the decision-making structure of ANFIS is

**Figure 5:**
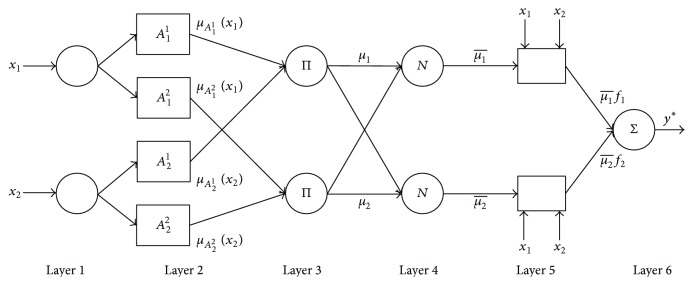
ANFIS model architecture.

(2) Rj:IF  x1  is  A1j  AND  x2  is  A2j  AND⋯AND  xn  is  Anj  THEN  y=fj=a0j+a1jx1+⋯+anjxn,

where *x*_*i*_ is the input variable, *y* is the output from the system, *A*_*i*_^*j*^ is the linguistic term with a corresponding membership function *μ*_*A*_*i*_^*j*^_(*x*_*i*_), and *a*_*i*_^*j*^ ∈ *R* are real coefficients. An example of ANFIS with two input variables and two decision rules is illustrated in Figure 5.

The architecture shown in Figure 5 is in the form of consecutive activities of six layers. The first layer has no function other than distributing input variables to linguistic terms. *A*_*i*_^*j*^ denotes the fuzzy set responsible for variable *x*_*i*_, which is featured in rule *R* ^*j*^ and defined by the corresponding membership function *μ*_*A*_*i*_^*j*^_(*x*_*i*_). The value of the membership function is generated at the output from these blocks. The third layer contains blocks denoted by Π, which are purely multipliers. In order to design a general structure, the values of rule triggers need to be normalized, such that their sum is equal to 1. This is the task of the fourth layer, whose blocks are denoted by *N* and where normalization takes place. The fifth layer computes the weighted consequent value. Finally, the sixth layer comprises only one block, denoted by Σ, where the weighted consequent values generated by all the rules are added up.

A very important feature of such a reasoning system is that its parameters can be tuned by the backpropagation error method. Additionally, consequent parameters can also be estimated by Kalman filtration. In other words, well-known and frequently used parameter tuning or estimation techniques can be used in this case as well.

The ANFIS model used in the present research was similar to a special three-layer feedforward neural network. The first layer represented the input variables, the hidden layer represented the fuzzy rules, and the third layer was the output.

In order to arrive at a fair comparison between neural networks and ANFIS, the ANFIS used had to have a number of tunable parameters, as similar as possible to the designed neural network (Table 3). The complexity of ANFIS can readily be influenced by the selection of the cluster radius, specified at initial clustering by the subtractive clustering method. The value of this parameter can be taken from the interval [0,1] and, roughly speaking, it represents the cluster radius in the input vector normalized space. If a higher value of this parameter is selected, the resulting ANFIS will have fewer membership functions of the input variables and fewer fuzzy rules. In the simulations discussed below, this parameter was selected such that each variable was represented by two membership functions; there were two fuzzy rules and the ANFIS output function was linear. This resulted in an ANFIS with 68 parameters to be tuned, the number that was closest to the total number of unknown parameters (40), which need to be tuned in neural networks with three nodes in the hidden layer. A membership function of the Gaussian type and a hybrid training method were used. The hybrid method was a combination of the backpropagation error and least squares methods. The results, regarding the number of parameters that had to be tuned, are shown in Tables 3, 4, and 5.

## 4. Results and Discussion

In order to make a consistent comparative analysis between the considered models, it was necessary to adopt the appropriate evaluation criteria [10, 50]. The used statistical model-performance indicators were root mean squared error (RMSE), coefficient of determination (*R*), and coefficient of efficiency (COE). RMSE is generally used as a measure of the difference between values predicted by the model and real, measured values. RMSE is an indicator of model accuracy or precision. RMSE should be as low, or as close to zero, as possible. The coefficient of determination *R* establishes a linear correlation between measured values and values simulated by the model. The optimal value is 1.

The Nash-Sutcliffe coefficient of efficiency (COE) of the model is used to assess (estimate) the accuracy of a model forecast. The interval of this coefficient is from −*∞* to 1. The value 1 corresponds to a situation where simulated values match measured values perfectly. If the coefficient of efficiency is equal to 0 (*E* = 0), then the accuracy of the model prediction is the same as that of the mean values of the measured data. Consider

(3) RMSE=∑i=1Nyio−yic2N,R=∑i=1Nyio−y−oyic−y−c∑yio−y−o2∑i=1Nyic−y−c2,COE=1−∑i=1Nyio−yic2∑i=1Nyio−y−2,

where *y*_*i*_^*o*^ and *y*_*i*_^*c*^ are the observed and calculated values at time *t*, respectively, and *y* ^*o*^ and *y* ^*c*^ are the mean of the observed and calculated values.

Tables 4 and 5 contain the values of criteria generally used to evaluate models of physical processes. RMSE, *R*, and COE were used in the present research: Table 4 relates to neural networks, while Table 5 depicts the same parameters for ANFIS. ANN used backpropagation errors, while ANFIS applied a hybrid method (a combination of least squares and backpropagation error). It is apparent from these tables that both models can be used to model water tables with a high level of accuracy and that there is no significant effect of the distance of the well from the river channel, given that model precision, expressed via RMSE, was roughly the same in all three cases: 0.15248, 0.14154, and 0.15029, respectively, with ANN, and 0.15097, 0.14756, and 0.15239, respectively, with ANFIS. These values were much lower than those predicted 2, 3, 4, 5, and 6 months in advance by Nayak et al. [51] and similar to one-month forecasts by means of LM propagation (Krishna et al. [8] and Maheswaran and Khosa [47]). An analogous conclusion follows from a comparison of *R* and COE. In the case of ANN, *R* varied from 0.92363 to 0.9615 and with ANFIS from 0.91973 to 0.9623. Mohanty et al. [10] reported similar values of *R*, but higher values of RMSE, for one-week water table forecasts relating to a river island in a tropical humid region in India.

Nash-Sutcliffe efficiency varied from 0.8510 to 0.9244 with ANN and from 0.84588 to 0.92586 with ANFIS. For these parameters to be comparable and for the comparison to be fair, the present research sought to ensure that the numerical complexity of the two modeling approaches was similar. Given that the ANN and ANFIS architectures were not the same, the main complexity quantifier was the number of unknown parameters that needed to be tuned through model training. The numerical values shown in the tables suggest that ANN and ANFIS are almost equally effective for physical modeling of depths-to-water table. The differences between the evaluation criteria are negligibly small (in the permille range). The authors gained additional experience (which could not be reported here due to paper length constraints) that the results could be improved further but still remain comparable, if more hidden nodes were added to ANN or the cluster radii reduced in ANFIS. These findings are consistent with those of Affandi and Watanabe [37] and Moosavi et al. [36] who compared ANFIS and two ANN algorithms, namely, Levenberg–Marquardt (LM) and the radial basis function (RBF), and concluded that there was no significant difference between the techniques used.

**Figure 6:**
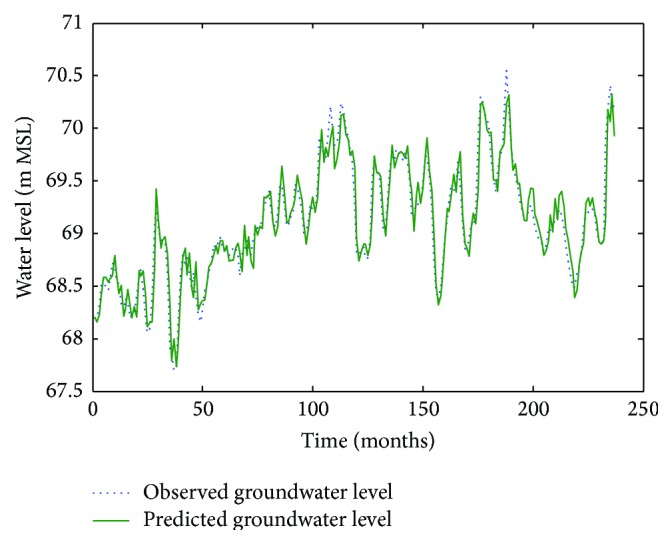
Comparison of observed groundwater levels with simulated results for 1 month ahead using an ANN trained with the Levenberg–Marquardt algorithm (Pp 930).

**Figure 7 fig7:**
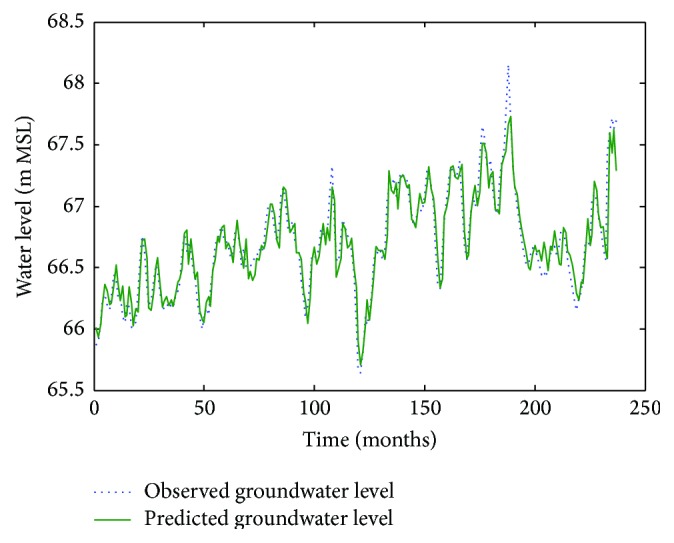
Comparison of observed groundwater levels with simulated results for 1 month ahead using an ANN trained with the Levenberg–Marquardt algorithm (Lp 927).

**Figure 8:**
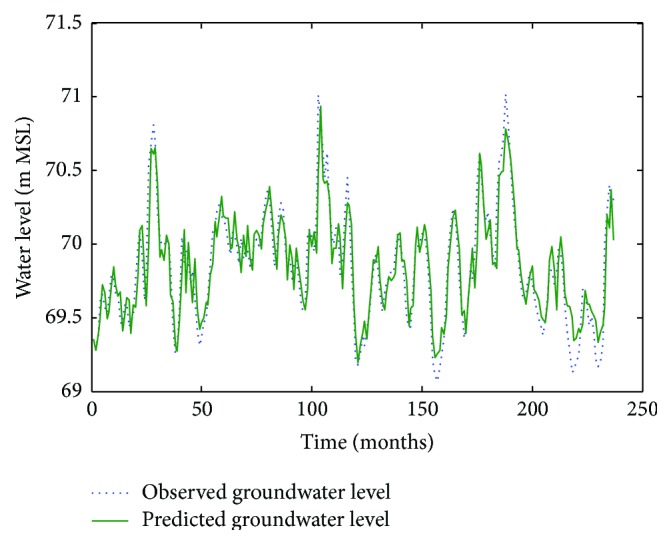
Comparison of observed groundwater levels with simulated results for 1 month ahead using an ANN trained with the Levenberg–Marquardt algorithm (Pp 928).

**Figure 9:**
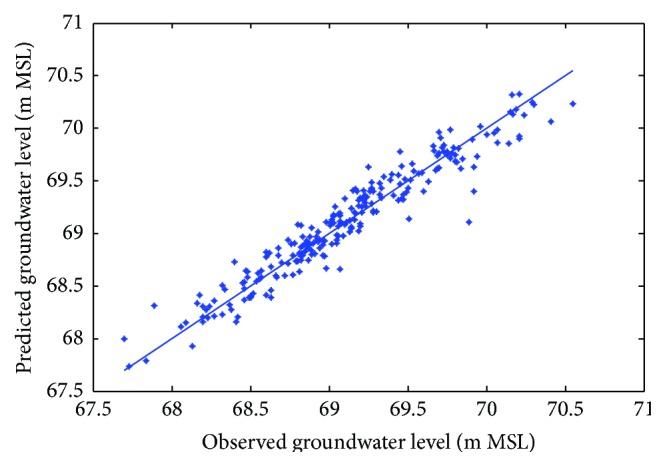
Scatter plots of observed and predicted groundwater levels using an ANN trained with the Levenberg–Marquardt algorithm (Pp 930).

**Figure 10 fig10:**
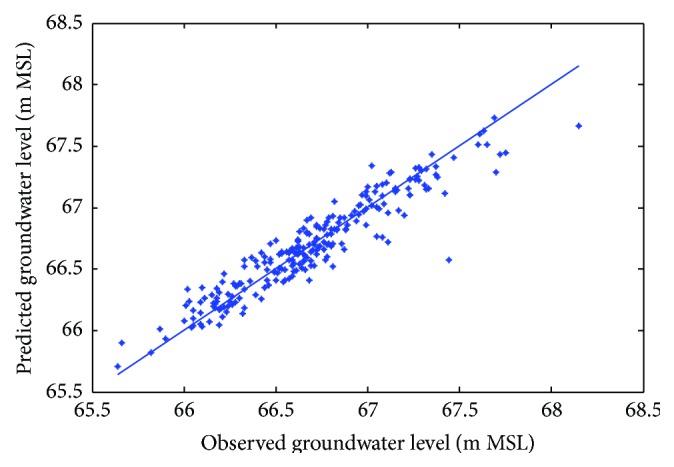
Scatter plots of observed and predicted groundwater levels using an ANN trained with the Levenberg–Marquardt algorithm (Lp 927).

**Figure 11:**
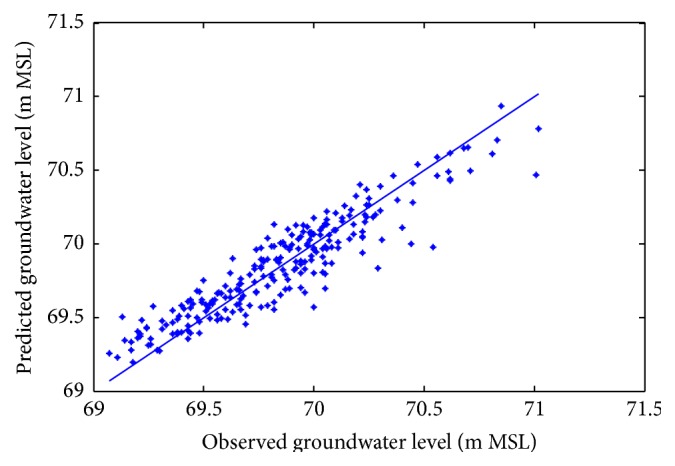
Scatter plots of observed and predicted groundwater levels using an ANN trained with the Levenberg–Marquardt algorithm (Pp 928).

**Figure 12:**
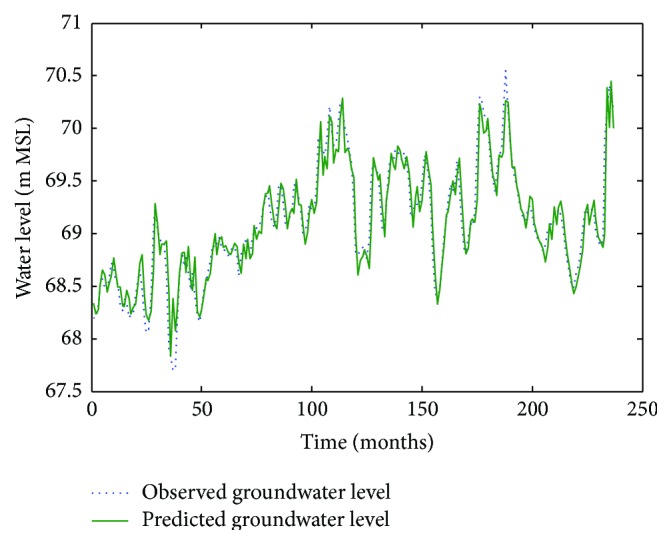
Comparison of observed groundwater levels with simulated results for 1 month ahead using an ANFIS model (Pp 930).

**Figure 13 fig13:**
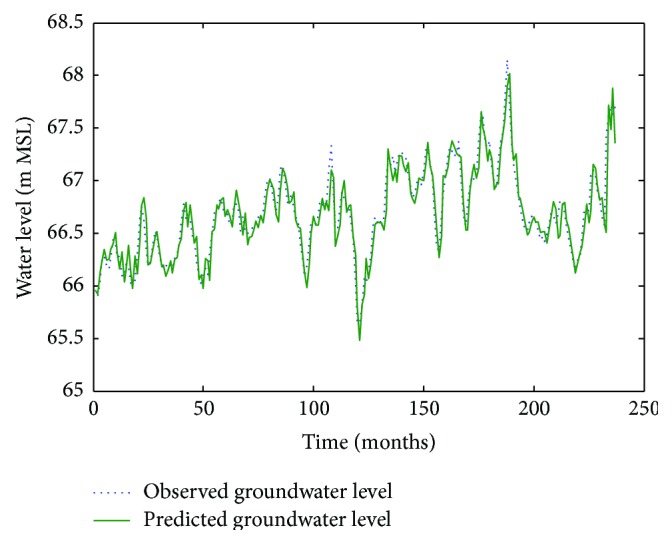
Comparison of observed groundwater levels with simulated results for 1 month ahead using an ANFIS model (Lp 927).

**Figure 14 fig14:**
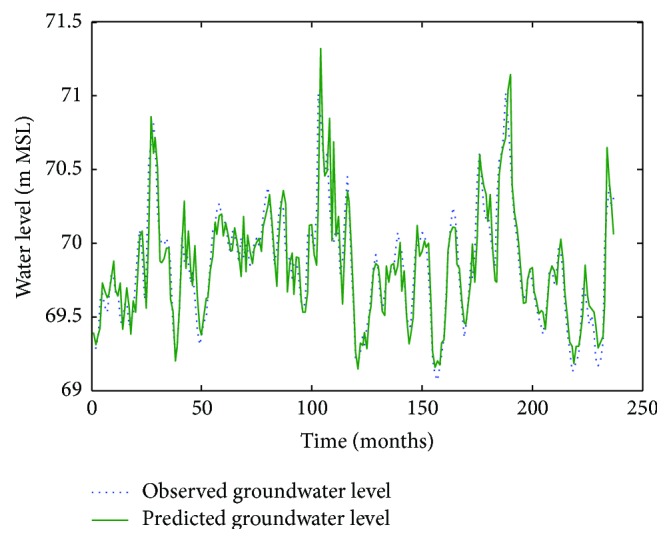
Comparison of observed groundwater levels with simulated results for 1 month ahead using an ANFIS model (Pp 928).

**Figure 15 fig15:**
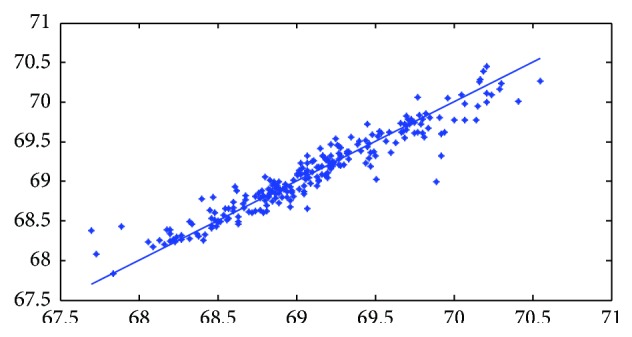
Scatter plots of observed and predicted groundwater levels using an ANFIS model (Pp 930).

**Figure 16 fig16:**
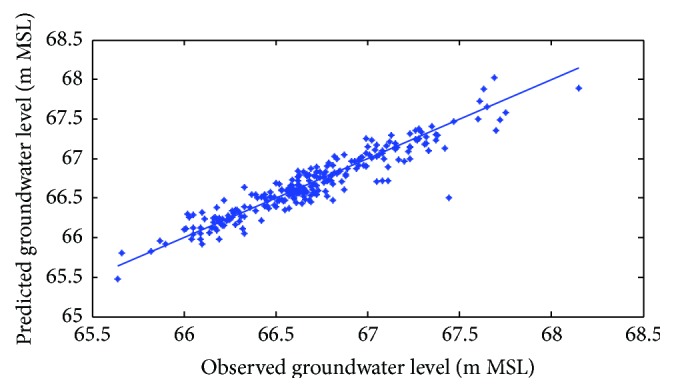
Scatter plots of observed and predicted groundwater levels using an ANFIS model (Lp 927).

**Figure 17:**
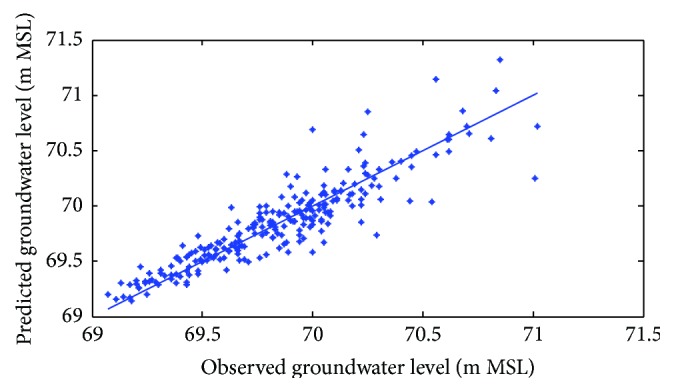
Scatter plots of observed and predicted groundwater levels using an ANFIS model (Pp 928).

Figures 6 [Fig fig7]–8 show measured versus monthly depths-to-water table predicted by the ANN model. Figures 9 [Fig fig10]–11 show scattered plots of values predicted by the same model, along with actual depths-to-water table. All the data points in Figures 9–11 are two-dimensional. The first coordinate shows the observed values and the second the predicated values. The coordinates are labeled accordingly. Thereafter, Figures 12 [Fig fig13] [Fig fig14] [Fig fig15] [Fig fig16]–17 show the same for the ANFIS model, respectively.

Such results undoubtedly lead to a significant inference, that both ANN and ANFIS are very useful tools for modeling hydrological processes in agriculture and that their computing and memory capabilities are similar, but expert judgment of the users of these tools is still of overriding importance in terms of defining the set of input parameters and the training set. If the physical variables are well selected and if they truly reflect the causal relationships of the physical processes (dependency of water table on river stages), ANN and ANFIS equally represent an extremely good numerical framework for generating high-quality models.

## 5. Conclusion

The results reported in this paper relate to the riparian lands along the left bank of the Danube River, in the Province of Vojvodina (northern part of Serbia). Two soft computing techniques were applied: adaptive neurofuzzy inference system (ANFIS) and feed-forward neural network with a Levenberg–Marquardt (LM) algorithm, to predict depths-to-water table one month in advance, at three wells located at different distances from the river.

Both models could be used with a high level of precision to model depths-to-water table, with no significant effect of the distance between the well and the river, as model precision expressed via RMSE was roughly the same in all three cases.

The following performance criteria were applied to compare the ANN and ANFIS models: root mean squared error (RMSE), coefficient of determination (*R*), and coefficient of efficiency (COE). To ensure that the comparison was valid, the complexity of the two models (ANN and ANFIS) was made comparable. The complexity was quantified through the number of unknown parameters that needed to be tuned during the course of training. The conclusion of the research is that both models can be used with a high level of precision to model water tables without a significant effect of the distance of the well from the river, as model precision expressed via RMSE was roughly the same in all three cases (0.14154–0.15248). *R* varied from 0.91973 to 0.9623, and COE from 0.84588 to 0.92586. The results suggest that both techniques are useful tools for modeling hydrological processes in agriculture, that their computing and memory capabilities are similar, and that they represent an exceptionally good numerical framework for generating high-quality models.

## Figures and Tables

**Table 1:** The coefficient of correlation between groundwater levels temperature (*T*max, *T*min), evapotranspiration (ETo), and precipitation (*P*).

Groundwater levels Well	*T*max	*T*min	ETo	*P*
Pp 930	0.001	−0.006	0.06	0.022
Lp 927	0.002	0.001	0.002	0.037
Pp 928	0.016	0	0.001	0.006

**Table 2:** Variables in the input vector to ANN and ANFIS models.

Pp 930			
Pp 930	Pp 930 (*t*-1)	Pp 930 (*t*-2)	Pp 930 (*t*-3)
Lp 927	Lp 927 (*t*-1)	Lp 927 (*t*-2)	Lp 927 (*t*-3)
Pd 15	Pd 15 (*t*-1)	Pd 15 (*t*-2)	Pd 15 (*t*-3)
Danube	D (*t*-1)		
Channel	C (*t*-1)		

Lp 927			
Lp 927	Lp 927 (*t*-1)	Lp 927 (*t*-2)	Lp 927 (*t*-3)
Pp 930	Pp 930 (*t*-1)	Pp 930 (*t*-2)	Pp 930 (*t*-3)
Pd 7	Pd 7 (*t*-1)	Pd 7 (*t*-2)	Pd 7 (*t*-3)
Danube	D (*t*-1)		
Channel	C (*t*-1)		

Pp 928			
Pp 928	Pp 928 (*t*-1)	Pp 928 (*t*-2)	Pp 928 (*t*-3)
Lp 927	Lp 927 (*t*-1)	Lp 927 (*t*-2)	Lp 927 (*t*-3)
Pp 930	Pp 930 (*t*-1)	Pp 930 (*t*-2)	Pp 930 (*t*-3)
Danube	D (*t*-1)		
Channel	C (*t*-1)		

**Table 3:** ANFIS model parameters.

	Pp 930	Lp 927	Pp 928
Number of nodes	62	62	62
Number of linear parameters	24	24	24
Number of nonlinear parameters	44	44	44
Total number of parameters	68	68	68
Number of training data pairs	164	152	147
Number of checking data pairs	73	85	90
Number of fuzzy rules	2	2	2

**Table 4:** ANN model performance criteria: root mean squared error (RMSE), coefficient of determination (*R*), and coefficient of efficiency (COE).

Well	RMSE	*R*	COE	Number of epochs	Best epoch
Pp 930	0.15248	0.9615	0.9244	55	49
Lp 927	0.14154	0.94316	0.8886	9	3
Pp 928	0.15029	0.92363	0.8510	13	7

**Table 5:** ANFIS model performance criteria: root mean squared error (RMSE), coefficient of determination (*R*), and coefficient of efficiency (COE).

Well	RMSE	*R*	COE	Number of clusters	Radii
Pp 930	0.15097	0.9623	0.92586	2	0.9
Lp 927	0.14756	0.93764	0.87893	3	0.9
Pp 928	0.15239	0.91973	0.84588	2	0.9
